# Immigration-Related Trauma Associated With Metabolic Risk and Cognition in Hispanic and Latino Immigrant Populations

**DOI:** 10.1093/geroni/igab046.291

**Published:** 2021-12-17

**Authors:** Erica Diminich, Kristine Ajrouch, Toni Antonucci, Sean Clouston, Irving Vega, Laura Zahodne, Noah Webster, Richard Gonzalez

**Affiliations:** 1 Stony Brook University, Stony Brook, New York, United States; 2 University of Michigan, Ypsilnati, Michigan, United States; 3 University of Michigan, Ann Arbor, Michigan, United States; 4 Renaissance School of Medicine, Stony Brook University, Renaissance School of Medicine, Stony Brook University, New York, United States; 5 Michigan State University, Grand Rapids, Michigan, United States

## Abstract

Recent immigrant and undocumented Hispanic/Latino adults in the United States (U.S.) are an underserved segment of the aging population. In this cross-sectional pilot study, we examined associations between self-reported stressors metabolic syndrome, emotional reactivity, and cognitive functioning in a heterogenous sample (N=80) of Hispanic/Latino adults (43.8% Central America; 43.8% South America; 7.5% Caribbean; mean years in the U.S.=18.1, SD=12.8). Participants (Meducation=10.2 years, SD=5.34; Mage=48.6 years, SD=12.3) underwent blood draw, anthropometrics and NIH-toolbox cognitive and behavioral measures. Linear regressions indicated that, elevated glucose was inversely associated with working memory (r=-.30), whereas higher HDL and controlled glucose were associated with better episodic memory (r=.27) and executive functioning (r=.32). Results further revealed associations between immigration-related trauma and elevated posttraumatic stress symptomatology. Implications for mental health and early detection of modifiable risk factors to promote healthy aging in vulnerable Hispanic/Latino immigrant populations are discussed.

